# Nox2 Activity Is Required in Obesity-Mediated Alteration of Bone Remodeling

**DOI:** 10.1155/2018/6054361

**Published:** 2018-11-08

**Authors:** Md Mizanur Rahman, Amina El Jamali, Ganesh V. Halade, Allal Ouhtit, Haissam Abou-Saleh, Gianfranco Pintus

**Affiliations:** ^1^Department of Biological and Environmental Sciences, College of Arts and Sciences, Qatar University, PO Box 2713 Doha, Qatar; ^2^Division of Nephrology, Department of Medicine, University of Texas Health Science Center at San Antonio, 7703 Floyd Curl Drive, Texas 78229-3900, USA; ^3^Division of Cardiovascular Disease, Department of Medicine, University of Alabama at Birmingham, Birmingham, Alabama 35294, USA; ^4^Department of Biomedical Sciences, College of Health Sciences, Qatar University, PO Box 2713 Doha, Qatar; ^5^Department of Biomedical Sciences, College of Medicine, University of Sassari, 07100 Sassari, Italy; ^6^Biomedical Research Center, Qatar University, Doha, Qatar

## Abstract

Despite increasing evidence suggesting a role for NADPH oxidases (Nox) in bone pathophysiology, whether Nox enzymes contribute to obesity-mediated bone remodeling remains to be clearly elucidated. Nox2 is one of the predominant Nox enzymes expressed in the bone marrow microenvironment and is a major source of ROS generation during inflammatory processes. It is also well recognized that a high-fat diet (HFD) induces obesity, which negatively impacts bone remodeling. In this work, we investigated the effect of Nox2 loss of function on obesity-mediated alteration of bone remodeling using wild-type (WT) and Nox2-knockout (KO) mice fed with a standard lab chow diet (SD) as a control or a HFD as an obesity model. Bone mineral density (BMD) of mice was assessed at the beginning and after 3 months of feeding with SD or HFD. Our results show that HFD increased bone mineral density to a greater extent in KO mice than in WT mice without affecting the total body weight and fat mass. HFD also significantly increased the number of adipocytes in the bone marrow microenvironment of WT mice as compared to KO mice. The bone levels of proinflammatory cytokines and proosteoclastogenic factors were also significantly elevated in WT-HFD mice as compared to KO-HFD mice. Furthermore, the in vitro differentiation of bone marrow cells into osteoclasts was significantly increased when using bone marrow cells from WT-HFD mice as compared to KO-HFD mice. Our data collectively suggest that Nox2 is implicated in HFD-induced deleterious bone remodeling by enhancing bone marrow adipogenesis and osteoclastogenesis.

## 1. Introduction

Obesity is defined as a body mass index (BMI) greater than or equal to 30. The obesity epidemic correlates with increasing evidence that lipotoxicity and inflammation might be the cause of bone loss [1–3]. Studies in mice and humans indicate that obesity is related to a sustained and elevated inflammatory state of adipose tissue [3, 4]. Several molecular and cellular determinants involved in inflammation- and lipotoxicity-induced bone mass loss have been suggested [1, 5]. Obesity is associated with enhanced tissue inflammation [6]. More recently, it has been shown that a fat increase in the bone marrow (BM) microenvironment [7] may affect bone homeostasis, by inhibiting osteoblast function and increasing osteoclast differentiation/activation [8], through the regulation of inflammatory cytokines [9]. Nevertheless, data concerning the impact of obesity on bone health are still controversial. Indeed, the augmented body mass due to obesity may increase mechanical bone stretching improving both bone mass and mineral density, while increased adiposity in the bone marrow region can lower osteoblast/osteocyte formation, generating low-quality bone and enhanced risk of fracture. However, some studies showed that obesity reduces fracture risk and protects against osteoporosis in adults [10, 11], while others showed that obesity does not protect against fracture in postmenopausal women [12, 13]. Interestingly, one study reported that obesity is a risk factor for fracture in children, while it is protective against fracture in adults [14], suggesting that the effects of obesity on bone parameters may differ with age.

Akin to obesity, a high-fat diet (HFD) can influence the bone microenvironment and remodeling. For instance, decreases in both bone resorption and formation have been reported in HFD-fed mice [15], while increased bone mineral density and decreased osteoclast activity have been shown in rats [16]. We have demonstrated that HFD can promote inflammation of the bone microenvironment and negatively influence bone remodeling toward osteoclastogenesis in mice [17]. The different reported effects in these studies might be due to differences in diet composition and animal models used. Indeed, decreased bone resorption and bone formation have been reported in patients with diabetes [18, 19] suggesting that both diet- and metabolic-related factors may differentially affect the bone microenvironment. It has been shown that knockdown of Nox2 can ameliorate the adipose tissue inflammatory status in HDF mice [20, 21]. In addition, inflammatory cytokines such as tumor necrosis factor alpha (TNF-*α*) can activate Nox2 and increase its activity [22, 23]. Therefore, Nox2 and inflammation are directly interrelated since enhanced inflammation in the bone microenvironment can negatively affect bone remodeling toward bone resorption. Therefore, we may assume that nox2 has a strong role in obesity-mediated bone remodeling.

Oxidative stress is shown to be a key mediator of bone loss [24, 25]. Elevated levels of reactive oxygen species (ROS), specifically superoxide, are consistently associated with increased osteoclastic activity in patients with bone disorders [26, 27] such as bone loss observed in the elderly population or patients with rheumatoid arthritis. The age-associated decrease in sexual hormones in both men and women is also an important risk factor for bone loss that is related to a decrease in sexual hormones. Finally, it is commonly observed that elderly osteoporotic patients are deficient in vitamins with antioxidant properties [27]. Whether the bone loss is due to a ROS overproduction and/or to an antioxidant level reduction, the resulting oxidative stress plays an essential role in osteoporosis. Given the complexity of the various sources of ROS in the bone and the etiology of bone disorders, the identification of the source of ROS for each disease and the elucidation of the mechanism by which ROS are produced and contribute are essential to developing specific and efficient therapy [25, 28].

NADPH oxidases (Nox) are considered key ROS-generating enzymes, whose activity is essential for normal cell function [29]. The Nox family encompass the gp91phox (Nox2), Nox1, Nox3, Nox4, Nox5, Duox-1, and Duox-2. In addition to Nox2, Nox1 and Nox4 are also known to be expressed in the bone microenvironment [25]. However, in this present work, we have mainly studied the role of Nox2 in obesity-mediated bone remodeling using a Nox2-knockout mouse model. In the bone microenvironment, hematopoietic stem cells commit to bone resorbing osteoclastic lineage in response to the receptor activator of NF-*κ*B ligand (RANKL) which is produced by osteoblasts, osteocytes, and bone marrow stromal cells [30]. The ligand-mediated activation of the RANK receptor by RANKL triggers a Nox-derived ROS-dependent signaling cascade which is crucial for osteoclast differentiation [31]. In particular, Nox1 and Nox2 are reported to be implicated in osteoclast differentiation in response to RANKL activation, while Nox4 and Nox2 have been reported to contribute to the bone resorption activity of mature osteoclasts [32–35]. By contrast, whether Nox enzymes are involved in osteoblast differentiation and function is still under investigation [36, 37]. In this context, some evidence suggests the implication of Nox4 in osteoblast differentiation and that of Nox2 in osteoblast precursor cell proliferation [25]. However, these findings were not confirmed in vivo in knockout mice.

Studies investigating the role of oxidative stress in bone diseases have been mainly performed by determining the effect of ROS overproduction on bone cell function and bone remodeling. It has been reported that ROS can promote bone loss by lowering osteoblast differentiation and inducing their apoptosis while concomitantly stimulating RANKL-induced osteoclast formation [38]. In addition, an osteoclast-generated superoxide increase is involved in bone matrix degradation [39]. While we showed that the expression level of cathepsin K, which is an indicator of bone resorption activity, is increased by a HFD in a redox-dependent manner [40], the exact role of Nox2 as a major source of ROS remains unknown.

In the present study, we focused upon investigating the role of Nox2 in obesity-mediated inflammation-dependent bone remodeling. Our results show that a HFD augments bone marrow adipogenesis and osteoclastogenesis in a Nox2-dependent manner.

## 2. Materials and Methods

### 2.1. Reagents and ELISA Kits

*α*-Modified minimal essential medium (*α*-MEM), Roswell Park Memorial Institute (RPMI) 1640 medium, phenol red-free *α*-MEM, Hanks' balanced salt solution (HBSS), and fetal bovine serum were purchased from Sigma-Aldrich (St. Louis, MO, USA). Recombinant mouse RANKL and M-CSF were obtained from PeproTech Inc. (Rocky Hill, NJ) and R&D Systems (Minneapolis, MN, USA), respectively. Phorbol 12-myristate 13-acetate (PMA) and superoxide dismutase (SOD) were obtained from Sigma Chemical (St. Louis, MO, USA). The enhancer-containing luminol-based detection system (Diogenes) was obtained from National Diagnostics (Atlanta, GA, USA). Experiments were performed following our previously published procedures as reported in Halade et al. [17].

### 2.2. Animals and Diet

Six-week-old wild-type (WT) and Nox2-knockout (KO) male mice were purchased from Jackson Laboratories (Bar Harbor, Maine 04609, USA) and provided water and standard American Institute of Nutrition (AIN) 93G (a diet recommended by AIN for growth) ad libitum. At 8 weeks, age-matched animals were randomized into two groups, each containing 10 mice. Subsequently, the mice were housed in a standard controlled animal care facility in cages (5 mice/cage) and fed with a high-fat diet (Jackson lab. DIO diet 45% fat; HFD) or a standard diet (SD) ad libitum for 3 months. National Institutes of Health guidelines were strictly followed, and all the studies were approved by the Institutional Laboratory Animal Care and Use Committee of the University of Texas Health Science Center (San Antonio, TX). Body weight was measured weekly. After completion of the 5-month period, animals from both experimental groups were sacrificed under isoflurane anesthesia. Experiments were performed following our previously published procedures as reported in Halade et al. [17].

### 2.3. Measurement of Bone Mineral Density (BMD)

Mice were anesthetized with ketamine/xylazine, and a dual-energy X-ray absorptiometry (DXA) scan was performed as described previously [41] before (mice aged 8 weeks) and after the administration of the diet for 3 months (mice aged 20 weeks) using a Lunar PIXImus mouse bone densitometer (General Electric). BMD was analyzed manually with PIXImus software as described previously [41].

### 2.4. Preparation and Culture of Primary Bone Marrow (BM) Cells and Osteoclast Differentiation

At the conclusion of the study, BM cells were isolated from the tibiae and femurs of all groups of mice according to our previously described methods [17, 42]. Briefly, isolated BM cells were cultured in *α*-MEM containing 10% heat-inactivated fetal calf serum (FCS; Invitrogen), 100 U/ml penicillin G, and 100 *μ*g/ml streptomycin at 37°C for 2 h under 95% air and 5% HFD_2_. Nonadherent cells were carefully harvested and centrifuged at 2000 rpm for 5 min at room temperature, and viability was determined using the trypan blue exclusion method. They were subsequently cultured in *α*-MEM medium in 24-well (1 × 10^6^ cells/well) clear-bottom white culture plates, supplemented with or without 50 ng/ml RANKL and 20 ng/ml M-CSF. A half-volume of the medium, with or without RANKL/M-CSF, was replaced with the fresh medium every 3 days. At day 6, plates were fixed and the number of formed osteoclasts was determined by staining cells with tartrate-resistant acid phosphatase (TRAP) using a TRAP staining kit (Sigma, St. Louis, MO, USA). TRAP-positive multinucleated cells (MNCs) of more than three nuclei were counted as osteoclasts under microscopic observation and expressed as a number of cells per field.

### 2.5. Bone Histology

Left femur specimens from each group were collected and trimmed of excess tissue and were fixed in 10% neutral buffer formalin (NBF) for 48 hours at room temperature (RT). Bone specimens were decalcified in 10% EDTA in water for 2 weeks at RT and were then placed in 70% ETOH, processed and embedded in paraffin, and stained with hematoxylin and eosin (H&E) as previously described [17, 43].

### 2.6. Quantitative Real-Time Reverse Transcriptase PCR (RT-PCR)

Experiments were performed following our previously published procedures [17]. Briefly, right whole femurs were crushed under liquid nitrogen conditions using a Kinematica Tissue Pulverizer, and RNA was isolated using the RNeasy Mini Kit following the manufacturer's instructions (Qiagen, Valencia, CA). Total RNA concentration was assessed in NanoDrop™ 1000 spectrophotometer (Thermo Scientific, Wilmington, DE, USA). mRNA expression of genes encoding IL-6, TNF-*α*, RANKL, cathepsin K (ctsk), runt-related transcription factor 2 (RUNX2), peroxisome proliferator-activated receptor (PPAR-*γ*), and 18S was measured by real-time RT-PCR carried out using Taq polymerase and SYBR green dye (Applied Biosystems, Foster City, CA) and an ABI Prism 7900HT Sequence Detection System (Applied Biosystems). mRNA Ct values for these genes were normalized to the housekeeping gene 18S.

### 2.7. Measurement of Superoxide in Freshly Isolated BM Cells

As previously reported, the rate of superoxide production by BM cells was determined using a luminol-based chemiluminescent reagent (Diogenes, National Diagnostics, GA) that is specific to superoxide [17, 44]. The cells were washed with PBS and placed in Hanks' balanced salt solution (HBSS). For the assay, a 100 *μ*l aliquot of the Diogenes reagent was mixed with a maximum of 1 × 10^5^ cells and incubated at 37°C for 2–4 minutes. Superoxide generation was stimulated with PMA (50 ng/ml) in the presence or absence of SOD (20 *μ*g/ml). Chemiluminescence was measured every minute for up to 60 minutes using a microplate reader (BioTek Clarity™, BioTek Instruments Inc., VT, USA) and an integration time of 5 seconds.

### 2.8. Statistical Analysis

Data are presented as mean values ± SEM of 3–4 experiments using 4–6 mice/group. The statistical analysis employed for each experiment is reported in the figure legends.

## 3. Results

### 3.1. ROS Production in Bone Marrow Cells Isolated from WT and KO Mice

**Figure 1:**
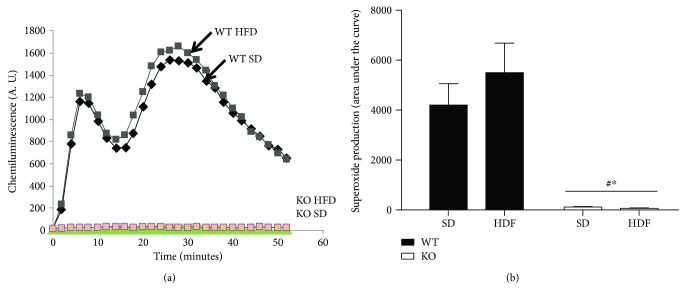
The high-fat diet does not alter superoxide production by the bone marrow cells. Superoxide production was measured using bone marrow (BM) cells isolated from wild-type and Nox2-knockout mice fed with either a standard (SD) or a high-fat diet (HFD) for 3 months. BM cells were stimulated with 50 ng/ml PMA, and chemiluminescence was monitored for the indicated time (a). After normalization by subtraction of the zero time value of the chemiluminescence output, the area *under the curve* was calculated as a measure of total superoxide production (b). Data are expressed as mean ± SEM (*n* = 4, 6 mice/group). Two-way ANOVA followed by post hoc Tukey's multiple comparison test was used to assess differences among groups with significance defined as *P* < 0.05. ^∗^Significantly different from SD-WT. ^#^Significantly different from HDF-WT.

Superoxide production was examined in freshly isolated bone marrow (BM) cells. A transient PMA-induced production of superoxide was observed in BM cells derived from WT mice. The observed superoxide generation reached its maximum in 5 minutes; then, it sustained up to 30 min and after that decreases to reach the nonstimulated levels in 60 to 90 min. The PMA-elicited superoxide production of BM cells was not significantly affected by the HFD (Figure 1). Abrogation of the chemiluminescence signals by the addition of the superoxide inhibitor superoxide dismutase (SOD) indicated that superoxide anion was specifically detected. As expected, BM cells isolated from KO mice were unable to produce any detectable amount of superoxide. These data indicated that a high-fat diet did not significantly alter superoxide production of BM cells and confirmed the phenotype of Nox2-knockout mice.

### 3.2. Effect of Nox2 on HFD Alteration of Bone Mineral Density (BMD)

**Figure 2:**
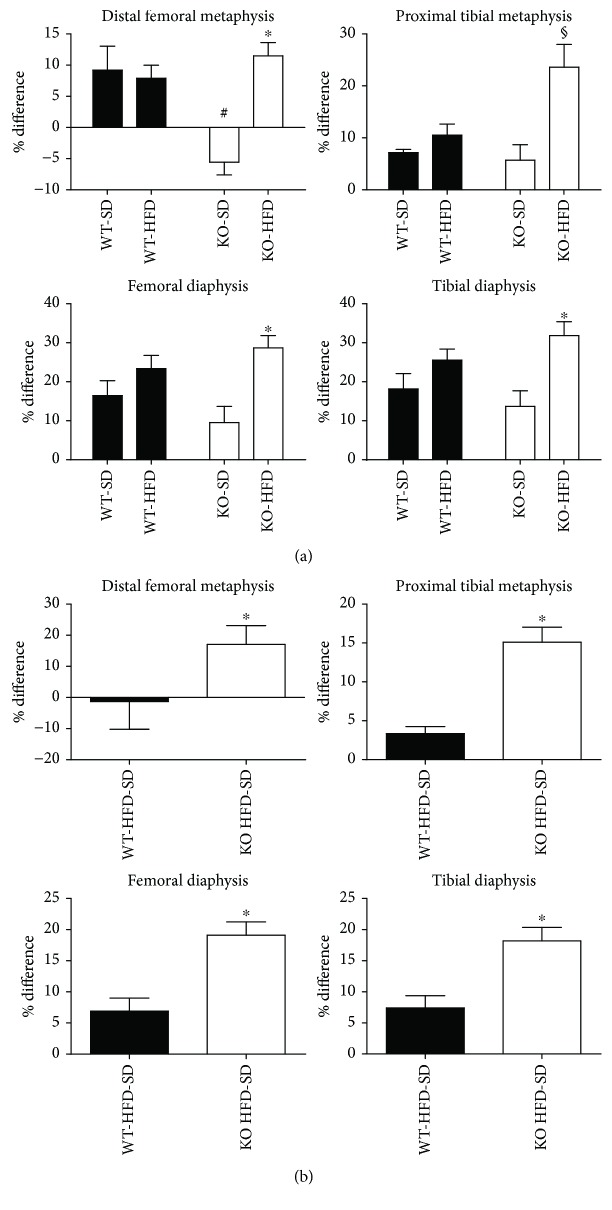
Effect of Nox2 on high-fat-diet- (HFD-) induced changes in bone mineral density (BMD). Eight-week-old WT and Nox2-KO mice were fed with either a standard lab chow diet (SD) or HFD for 3 months. Mice were scanned with dual-energy X-ray absorptiometry (DXA) before and after administration of experimental diets. (a) Percent changes in BMD before and after experimental diets in different bone regions. (b) Percentage change of the BMD difference between mice fed with an SD and an HFD in different bone regions. Data are expressed as mean ± SEM (*n* = 3, 4 mice/group). In (a), two-way ANOVA followed by post hoc Tukey's multiple comparison test was used to assess differences among groups with significance defined as *P* < 0.05. ^#^Significantly different from all the group data in the graph. ^§^Significantly different from all the group data in the graph. ^∗^Significantly different from SD-KO in all the graphs. In (b), unpaired *t*-test with Welch's correction has been used to assess significance between the groups with significance defined as *P* < 0.05. ^∗^Significantly different from each other in all the graph data.

Three months of feeding of a HFD significantly increased body weight and fat mass of both WT and KO mice. There was no significant difference in body weights (BW) and total fat mass (FM) between SD WT and SD KO mice (26.8 ± 0.44 g BW and 3.8 + 0.42 g total fat mass and 23.7 ± 1.5 g BW and 3.3 ± 0.24 g FM, respectively) and between HFD WT and HFD KO mice (41.3 ± 1.68 g BW and 8.6 ± 1.7 g FM and 36.3 ± 2.46 g BW and 9.9 + 2.3 g FM, respectively). We measured the bone mineral density of all four groups once before starting the experimental diets and again just before sacrifice. Bone mineral density was not significantly reduced in WT versus KO mice fed with a SD with the exception of the distal femoral metaphysis bone “section” (Figure 2(a)). An increase in bone mineral density was observed in both WT and KO animals in all bone sections except distal femoral metaphysis of HDF mice in comparison to that of SD mice (Figure 2(a)). However, the magnitude of this effect was significantly greater in KO mice as compared to WT mice as indicated by the data in Figure 2(b) reporting the percentage change of the difference between HDF and SD. Indeed, this figure clearly depicts that the differences in BMD between HFD- and SD-fed mice (Figure 2(a)) were significantly higher in KO mice as compared to WT mice indicating that HFD-induced BMD alteration depends on Nox2.

### 3.3. Nox2 Mediates HFD-Induced Bone Marrow Adiposity

**Figure 3:**
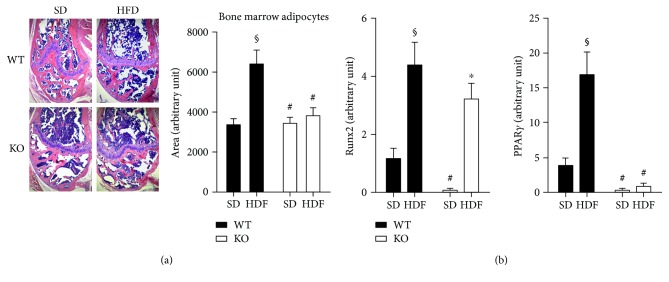
Effect of Nox2 on high-fat-diet- (HFD-) induced changes in bone marrow adiposity. Eight-week-old WT and Nox2-KO mice were fed with either an SD or an HFD for 3 months. (a) Left femurs were collected after sacrifice and fixed in 10% neutral-buffered saline (NBF). After processing, sections of bone were embedded in paraffin and stained with hematoxylin and eosin (H&E). Representative microphotographs of stained femur sections from each group are shown. Magnification ×100. The arrow shows adipocytes in the bone marrow cavity. Analysis of white areas (adipocytes) inside the bone marrow has been performed using NIH ImageJ software. (b) Right femurs were crushed under liquid nitrogen, and RNA was isolated and analyzed for mRNA levels for Runx2 and PPAR-*γ* by real-time RT-PCR. mRNA Ct values for these genes were normalized to the housekeeping gene 18S. Data are expressed as mean ± SEM (*n* = 3, 4 mice/group). Two-way ANOVA followed by post hoc Tukey's multiple comparison test was used to assess differences among groups with significance defined as *P* < 0.05. ^§^Significantly different from SD-WT. ^#^Significantly different from HFD-WT. ^∗^Significantly different from SD-KO.

Alteration of bone mineral density by a HFD is generally paralleled by enhanced bone marrow adiposity. We therefore asked whether Nox2 downregulation could affect bone marrow adiposity. Our results indicate that the HFD increased the number of adipocytes. Interestingly, this effect was dramatically reduced in KO mice (Figure 3(a)). In addition, we found that the expression level of PPAR-*γ*, a known marker of adipocyte differentiation and maturation, was increased in the bone of WT HFD mice as compared to WT SD mice (Figure 3(b)), and this effect was abrogated in KO mice. Since the same progenitor cells lead to the formation of osteoblasts and adipocytes, we measured the Runx2 expression level, a known marker of osteoblastogenesis. Interestingly, HFD significantly increased the osteoblastogenic factor Runx2 in both WT and KO mice. These data suggest that Nox2 regulates only bone marrow adiposity and does not alter osteoblastogenesis.

### 3.4. Nox2 Mediates HFD-Induced Osteoclastogenesis

**Figure 4:**
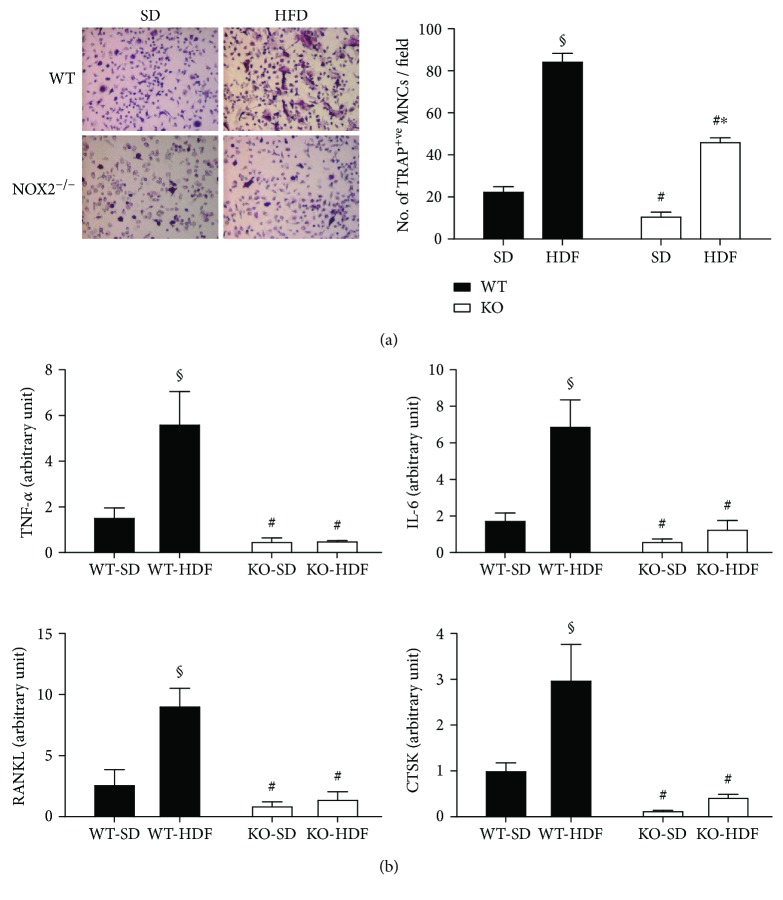
Effect of Nox2 on high-fat-diet- (HFD-) induced changes in bone marrow osteoclastogenesis. Eight-week-old WT and Nox2-KO mice were fed with an SD or an HFD for 3 months. (a) Bone marrow cells were isolated from the left femur and tibia of each group. Bone marrow cells (1 × 10^6^) were cultured in the presence of RANKL and MCSF for 5 days. Cells were then stained for TRAP and counted under a microscope. TRAP-positive multinucleated cells (MNCs) containing more than 3 nuclei were considered osteoclasts. Bars show the number of TRAP-positive MNCs in each group expressed as a number of cells per field. (b) Right femurs were crushed under liquid nitrogen, and RNA was isolated and analyzed for mRNA levels for TNF-*α*, IL-6, RANKL, and cathepsin K (CTSK) by qRT-PCR. mRNA Ct values for these genes were normalized to the housekeeping gene 18S. Data are expressed as mean ± SEM (*n* = 4, 5 mice/group). Two-way ANOVA followed by post hoc Tukey's multiple comparison test was used to assess differences among groups with significance defined as *P* < 0.05. ^§^Significantly different from WT-SD. ^#^Significantly different from WT-HFD. ^∗^Significantly different from SD-KO.

Herein, we wanted to investigate the potential involvement of Nox2 in osteoclastogenesis. To this end, in vitro osteoclastogenesis was induced in BM cells isolated from all the experimental groups by stimulation with RANKL and MCSF. Our data indicated that BM cells from WT HFD mice formed a significantly higher number of TRAP-positive osteoclast-like cells as compared to BM cells isolated from WT SD mice (Figure 4(a)). Interestingly, this HFD-associated effect was significantly reduced when BM cells isolated from KO animals were employed, suggesting the involvement of Nox2 in HFD-induced osteoclastogenesis (Figure 4(a)). These observations are in agreement with the finding that proinflammatory and proosteoclastogenic factors such as TNF-*α*, IL-6, RANKL, and CTSK mRNA levels were significantly increased in the bones of WT HFD mice but not in those of KO HFD mice (Figure 4(b)). These data support the hypothesis that Nox2 is implicated in the increased bone resorption associated with HFD. More importantly, its absence protects the mice against the Nox2-dependent deleterious effect elicited by HFD on the bone. Our data suggest that HFD-induced Nox2-dependent bone deterioration is associated with enhanced osteoclastogenesis.

## 4. Discussion

Using a corn oil diet in 12-month-old female mice, we previously reported that obesity increased osteoclast formation and lowered osteoblast formation directing the overall bone homeostasis toward bone resorption [17]. In the present work, our results indicate that obesity-mediated bone remodeling in favor of osteoclastogenesis can occur independently from either the aging process or the gender type [17, 45–47]. Our data demonstrated that the magnitude of the increase in BMD was significantly greater in KO mice as compared to WT mice suggesting Nox2 implication in HFD-induced bone alteration.

While the alteration in BMD measured in growing mice cannot be considered bone loss, our data revealed that the HDF-induced bone remodeling occurred in a Nox2-dependent manner. We found that a HFD was able to increase both the number of adipose cells and the expression of PPAR-*γ* in the bone marrow. Interestingly, these HFD-associated effects were significantly reduced in KO mice while body weight and fat mass gain were similar to those observed in wild-type mice. This observation supports the idea that more than body weight or fat mass, the localization of fat cell accumulation is primordial in contributing to tissue lipotoxicity [48–50].

Both adipocytes and osteoblasts are generated from mesenchymal stem cells. Therefore, it has been proposed that a HFD can alter bone marrow cellular composition resulting in an increased adipocyte number and decreased osteoblasts. This imbalanced bone marrow composition would decrease osteogenesis [7], thus reducing BMD and bone mass. Our data indicate that HFD was able to increase Runx2, suggesting that osteoblastogenesis is not reduced, but on the contrary increased by this feeding regime. We think that this could be a primary adaptive mechanism to maintain bone integrity when there is an increase in the total body weight. Indeed, this is in accord with the increase in bone mineral density we observe in HFD mice (Figure 2). More importantly, we observed that only the formation of bone marrow adipocytes was affected in Nox2-knockout mice. Also, the Nox2-dependent alteration of BMD could not be attributed to osteoblastogenesis dysregulation. Since adipocytes secrete proinflammatory cytokines favoring osteoclast formation [40], we examined whether a HFD could affect osteoclastogenesis. Our result highlighted that the expression of proinflammatory and proosteoclastogenic factors such as TNF-*α*, IL-6, RANKL, and CTSK was significantly increased in the bones of WT HFD mice as compared to KO mice.

We also analyzed the formation of osteoclasts in vitro using mouse-derived BM cells. RANKL activation is a pivotal step toward mature osteoclast differentiation [51, 52]. Importantly, NADPH oxidase-mediated superoxide production has been reported to execute a key role in osteoclastogenesis [33], and RANKL is known to stimulate ROS production [32]. Given their macrophage nature, all forms of osteoclasts express high amounts of Nox2 at both the protein and mRNA levels [32–34, 53]. However, osteoclasts also express Nox4 in their mature form and Nox1 in undifferentiated and differentiated preosteoclastic BM cells [35, 54]. Our data indicate that Nox2 is a major producer of ROS by BM cells and that it contributes to RANKL-mediated osteoclast formation in vitro. Interestingly, Nox1 was also shown to be essential in osteoclast differentiation stimulated by RANKL [32]. In this study, Nox1 silencing significantly reduced ROS production and differentiation induced by RANKL. Given that ROS are shown to be a key intermediate in osteoclastogenesis [55], this finding is surprising because not only is Nox2 highly expressed in BM cells but also its activation generates a far greater amount of ROS. The explanation for these discrepancies might relate to the possibility that Nox1 siRNA altered the expression of Nox2. In this regard, it has been reported that increased expression of different Nox enzymes may compensate the loss of particular isoforms in osteoclasts [32, 56, 57]. Furthermore, other studies suggest that Nox1 and Nox2 might compensate for one another for RANKL-mediated stimulation of osteoclast formation [33]. Finally, recent works demonstrate that Nox4 expression is increased in Nox2-deficient osteoclasts and contributes to osteoclast formation [35, 54]. In accord with these in vitro studies, the absence of evident bone abnormality in Nox1-, Nox2-, and Nox4-deficient mice suggests that the genome redundancy can guarantee the ROS production that is necessary to maintain bone growth and homeostasis. In our study, we did not observe any significant effect of either the diet or the genotype on Nox1 and Nox4 mRNA expression levels (data not shown), suggesting that the effects observed in KO mice could reasonably be attributed to Nox2 deficiency.

In the present work, we observed that despite any gross alteration of the bone structure, WT KO mice tend to be smaller, and we observed an overall downregulation of genes controlling the bone environment (adiposity, osteoblast, osteoclast, and inflammation). However, our data reveal that under a pathological condition (i.e., HFD), Nox2 deficiency had a beneficial effect on bone. Nox2 deficiency counteracted HFD-induced bone marrow adiposity and osteoclastogenesis. Also, it is possible that the relevance and implication of each Nox enzyme in osteoclast formation and activity might be revealed only under a pathological or stress condition.

To our knowledge, the data reported in this study provide the first evidence that Nox2 mediates HFD-induced bone marrow adiposity and osteoclastogenesis without affecting osteoblastogenesis, therefore affecting bone homeostasis in favor of bone resorption.

## Data Availability

All data generated or analyzed during this study and used to support the current findings are included in the article.
